# Quality Traits of Dry-Cured Loins from Iberian Pigs Reared in *Montanera* System as Affected by Pre-Freezing Cure

**DOI:** 10.3390/foods10010048

**Published:** 2020-12-26

**Authors:** Alberto Ortiz, David Tejerina, Rebeca Contador, Ana Isabel de Andrés, María Jesús Petrón, Juan Manuel Cáceres-Nevado, Susana García-Torres

**Affiliations:** 1 Meat Quality Area, Center of Scientific and Technological Research of Extremadura (CICYTEX-La Orden), Junta de Extremadura, Ctra, A-V, Km372, 06187 Guadajira, Spain; alberto.ortiz@juntaex.es (A.O.); rebecontro@gmail.com (R.C.); garsus15@hotmail.com (S.G.-T.); 2 Food Technology, Agricultural Engineering School, University of Extremadura, Avda Adolfo Suárez s/n, 06007 Badajoz, Spain; aiandres@unex.es (A.I.d.A.); mjpetron@unex.es (M.J.P.); 3 Department of Animal Production, Faculty of Agricultural and Forestry Engineering, Agrifood Campus of International Excellence (ceiA3), University of Córdoba, Campus Rabanales, N-IV, km 396, Edificio de Producción Animal, 14014 Córdoba, Spain; caceresnevadojm@gmail.com

**Keywords:** pre-freezing cure, pre-frozen dry-cured loin, raw material, *Longissimus thoracis et lumborum*, *Montanera* seasonality

## Abstract

Iberian dry-cured loins from pigs fattened in *Montanera* are usually subjected to seasonal production, which could be overcome through freezing the loin before the curing process. Hence, three homogeneous batches (*n* = 15 per batch) were established to evaluate the effect of different pre-freezing cure (−20 ± 2 °C) times (three and six months) of raw material on main quality characteristics of dry-cured loins in comparison to those elaborated from unfrozen meat. All samples were subjected to similar seasoning and same curing length to obtain dry-cured loins. Results showed a decrease in lightness, redness, chrome and hue values, polyunsaturated fatty acids (PUFA), higher oxidative phenomena, and changes in rheological properties in fresh loins on account on freezing (*p* ≤ 0.05). Some quality parameters of dry-cured loins were affected by freezing, weight loss, and hardness being higher as well as salt content being lower. PUFA and Warner–Braztler Shear Force (*p* ≤ 0.05) also showed higher values in dry-cured loins from frozen meat than those elaborated from unfrozen counterparts.

## 1. Introduction

Iberian products enjoy great prestige worldwide due to their quality attributes, especially those which derive from pigs reared under *Montanera* system (outdoor final fattening phase based on acorns and grass) [1]. Nevertheless, considering the seasonality of *Montanera* system, dry-cured loins reach the market in summer, as a result of its short technological process (about 70 days) [2]. The highest consumption demand of these products takes place in November and December, during the Christmas season, this fact resulting in the existing time gap between industry production and consumer demand.

A growing practice among manufactures involves freezing raw material before its technological process of curing. It would allow manufacturers to balance production and demand, avoiding the fluctuations in the market price and subsequently, the seasonality could be overcome. On the contrary, the use of frozen/thawed counterparts as raw material could be detrimental to the inherent quality of the final product. Physical (water losses or changes on rheological properties) and chemical (colour or oxidative phenomena) changes in raw meat could have an impact on technological and nutritional attributes of dry-cured products [3]. Moreover, the use of frozen/thawed material could be a factor to be considered during seasoning and curing. Oxidation phenomena are of particular interest in this sense, since these are not completely inhibited during frozen storage. In fact, both protein [4] and lipid oxidation [5] are defined by [6] as the major forms of the deterioration in foods from muscle origin.

To date, scientific literature concerning to pre-freezing cure is scarce, and mostly focused on ham from commercial breed pigs [7,8]. Few studies deal with such a topic in hams from Iberian pigs [3,5,9], and only Lorido et al. [10] reported substantial contributions about the potential effect of pre-freezing cure on oxidative stability of the proteins of Iberian loins. Nevertheless, the evaluation of different freezing times has never been approached to the best of our knowledge. In addition, the aforementioned studies were carried out in meat from Iberian pigs fattened in confinement and commercially feeding system, even though the *Montanera* production system is the one where seasonality is an important issue, as has been previously explained. Abellán Ventanas, Akcan, and Estévez [11] focused on the effect of pre-freezing cure on proteolysis changes and sensory acceptance of loins but not from Iberian breed pigs.

On the other hand, some Specific Designations of Origin exclude the pre-freezing cure, whereas the current European regulation on freezing of food of animal origin [12] and the current Iberian Quality Standard (IQS) [2] make no mention to this practice. Thus, impact of this practice on the quality of the final product—dry-cured loin—remains unknown.

In order to generate scientific knowledge to help solving the seasonality of the production of dry-cured loin from Iberian pigs under the *Montanera* system, the objective of this study was to evaluate the effect of two freezing times (three and six months) on the quality characteristics of the raw material (fresh loins) and final quality characteristics of the dry-cured loins from Iberian x Duroc crossbred pigs under the *Montanera* system with respect to counterparts elaborated from fresh (unfrozen) ones.

## 2. Materials and Methods

### 2.1. Meat Sampling

A total of 45 *Longissimus thoracis et lumborum* muscles (LTL) were obtained from Retinto Iberian (Valdesequera line, Junta de Extremadura, Badajoz, Spain) crossed with Duroc (50%) pigs under the *Montanera* system. The rearing system conditions to which animals were subjected were as follows:

During growing period, from weaning to the beginning of *Montanera* (with a body weight (BW) of 102.9 ± 0.85 kg (mean ± standard deviation)) animals were housed in open-air corrals and fed based on commercial feeds. The average daily feed intake was 1.29 ± 0.08 kg/day, and the average daily gain (ADG) 256 ± 28.3 g/day. Afterwards, the finishing period in *Montanera*, was carried out in the Valdesequera farm, Junta de Extremadura, Badajoz, Spain, with a stocking rate of approximately 0.60 pigs per hectare. The length of the *Montanera* was of 67 days, from November 2018 to January 2019. *Montanera* feeding was based on ad libitum consumption of acorns mainly from *Quercus rotundifolia* and grass with free access to water. At the end of *Montanera*, animals had reached a BW of 148.4 ± 6.15 kg, with an ADG of 673 ± 34.9 g/day. The day previous to the slaughter animals were transported to a local slaughterhouse located 150 km approx. from the Valdesequera farm in a vehicle pen, where they were exposed to air flow during the transport, but they were not fed or watered. Animals were unloaded after 2-h journey approx. During lairage (less than 24 h) animals were provided of water whilst feed was withdrawn. Successively, animals were randomly slaughtered by exsanguination and previously stunned using a carbon dioxide stunning system.

**Figure 1:**
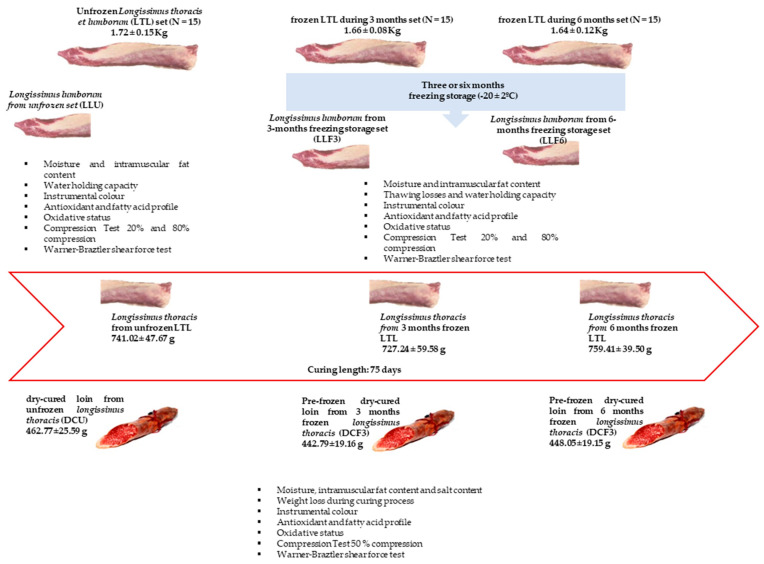
Experimental design.

After 4 h *post-mortem*, the LTL muscles were removed from the left side of the carcass and freed from intermuscular fat and connective tissue. Then, they were allowed to cool for 24 h at 4 ± 1 °C. Subsequently and previously to curing, the loins were randomly divided into three sets: unfrozen (*n* = 15) and pre-frozen dry-cured loin during 3 (*n* = 15) and 6 months (*n* = 15). The average weight of the LTL muscles were 1.72 ± 0.15, 1.66 ± 0.08 and 1.64 ± 0.12 kg, respectively. Each whole muscle (LTL) was divided into two parts: (i) the first portion corresponding to *Longissimus lumborum* muscle (LL) was used for fresh loin analyses (including those resulting from the freezing/thawing process) and (ii) the rest of the loin corresponding to the thoracic part—*Longissimus thoracis* (LT), was used for dry cured loin’s analyses (Figure 1).

### 2.2. Freezing and Thawing Process

The muscle *Longissimus lumborum* assigned to unfrozen loins (LLU) was subjected to analysis after cooling for 24 h. The remaining muscle samples (belonging to LT muscle) were cured, in order to obtain dry-cured loins (dry-cured loins from unfrozen pieces).

The rest of LTL muscles from pre-frozen dry-cured sets were individually packaged in nylon/polyethylene bags (O_2_ permeability, 9.3 mL O_2_/m^2^/24 h at 0 °C) and frozen during 3 or 6 months in a freezing room through cold air (−20 ± 2 °C) at 20 km/h. The freezing rate ranged between 1 and 5 cm/h, according to the common practice in meat industry. Thawing was carried out by storing loins at cooling temperature (4 ± 1 °C) for 48 h.

After thawing, a section of sample belonging to LL muscle was taken to evaluate the effect of freezing during three (LLF3) or six months (LLF6) on fresh loin. The remaining muscle samples (belonging to LT muscle) were immediately subjected to curing, in order to obtain dry-cured loins (pre-frozen dry-cured loins) (Figure 1).

### 2.3. Technological Process of Curing

Curing was carried out according to traditional meat processing industry standards though it was adapted to the weight of the loins (741.02 ± 47.67, 727.24 ± 59.58, and 759.41 ± 39.50 g for unfrozen and frozen during three and six months *Longissimus thoracis*, respectively). The processing conditions were homogenous in the three sets of manufactured dry-cured loins, (*n* = 15 unfrozen dry-cured loin (DCU) from unfrozen LT; *n* = 15 pre-frozen dry cured loin (DCF3) and *n* = 15 DCF6 from frozen during 3 and 6 months, respectively, and thawed LT muscle). Therefore, all loins were seasoned in the same way with a formula which contained nitrified salt (3%) and nitrites (0.8% of the total salt content) (percentages referred to total meat content) but garlic, paprika and olive oil, commonly used ingredients for seasoning of dry-cured loins were not used to avoid interferences with oxidative reactions. They were kept at a temperature of +4 °C for 48 h -in darkness, to allow the seasoning mixture to distribute into the meat. Subsequently, loins were stuffed into 10 cm diameter collagen casings and held at +4 °C and 75–80% relative humidity for 30 days. Then, loins were ripened at temperatures which ranged from 10 to 16 °C whereas HR decreased from 75 to 65% (45 days). Thus, the total curing length was 75 days [2]. Processing, seasoning, and curing length were similar in the current study, in order to evaluate the single effect of pre-freezing cure on quality traits of raw material and dry-cured loins.

Finally, when the curing process was concluded, dry-cured loins were vacuum-packed and kept in refrigeration until the corresponding analyses were carried out (less than one week). The average weight of the dry-cured loins was 462.77 ± 25.59, 442.79 ± 19.16 and 448.05 ± 19.15 g for DCU, DCF3 and DCF6 sets, respectively) (Figure 1).

### 2.4. Methods

#### 2.4.1. Proximate Composition

Moisture was assayed according to the AOAC method [13]. Intramuscular fat (IMF) was extracted with chloroform/methanol (2:1, *v/v*), following the Folch method [14]. In dry-cured loins chloride content (NaCl) was assayed, using Volhard method [15].

#### 2.4.2. Thawing Loss (TL) and Water Holding Capacity (WHC)

The difference between the weight of the sample before freezing and after thawing and expressed as thawing losses (TL) through the following equation:

% thawing losses = ((W_0_ − W_f_)/W_0_) × 100,

were W_0_ is the weight of sample (the whole muscle, LTL) before freezing and W_f_ the weigh after freezing and thawing process.

WHC (only in LL) was evaluated by measuring the water released of the sample after the application of a centrifugal force (3000 rpm during 3 min) following the method proposed by Tejerina et al. [16]

#### 2.4.3. Weight Loss in Dry-Cured Loins

Weight loss (WL) after curing was determined by gravimetric differences using the equation:

% weight loss = ((W_0_ − W_f_)/W_0_) × 100,

were W_0_ is the sample weigh (belonged to LT muscle) before seasoning and W_f_ the weigh at the end of the process (75 days).

#### 2.4.4. Colour Measurement

For both fresh and dry-cured loins, using a Minolta CR-400 colorimeter (Minolta Camera, Osaka, Japan) with illuminant D65, a 0° standard observer and a 2.5 cm port/viewing area using colour system CIE (L*a*b*) for determinate lightness (L*), redness (a*) and yellowness (b*) and the saturation index or chroma (C*), defined as (C = (a*^2^ + b*^2^) × 0.5) and Hue angle (H°) as arctg (b*/a*) were determined. The measurements were repeated at five randomly selected sites on each sample and averaged.

#### 2.4.5. Determination of α and γ-tocopherol

Antioxidants (α- and γ-tocopherol) content were assayed by the method proposed by Liu, Scheller, and Schaeffer [17]. Thus, tocopherol extraction was carried out with a saponifying solution (KOH 11.5% in EtOH/H2O 55:45), and determination was performed on an Agilent Technologies HPLC Series 1100 instrument (Agilent Technologies, Santa Clara, CA, USA) equipped with a Kromasil Silica column (5 µm particle size, 150 × 4.6 cm) (Symta, Madrid, Spain) and a Kromasil Silica Guard Column (10 µm) (Symta, Madrid, Spain). The mobile phase was hexane:isopropanol:etanol (98.5:1:0.5 *v/v*), at a flow rate of 1 mL/min and the fluorescence detector (Agilent Technologies Series 1200) was fixed at λ-excitation: 295 nm and λ-emission: 330 nm. Individual identification and quantification of the peaks were done by comparing with those of α- and γ-tocopherol authenticated commercial standards from Sigma-Aldrich in the 0.2–14 µg/mL range (Sigma-Aldrich, St. Louis, MO, USA).

#### 2.4.6. Lipid and Protein Oxidation

Lipid oxidation was assayed by the 2-thiobarbituric acid (TBA) method of Salih, Smith, Price & Dawson [18]. TBA-RS values were calculated from the standard (1,1,1,3-tetraethoxypropane, TEP) curve.

Protein oxidation was estimated by measuring the content of free thiol groups, spectrophotometrically at 412 nm and was calculated using an absorption coefficient of 13.6 mM-1 cm-1. Protein concentration was determined by spectrophotometry at 280 nm using bovine serum albumin (BSA) as standard according to Batifoulier, Mercier, Gatellier, and Renerre [19]. Measurements were made for each sample, and mean values were used for further statistical analysis.

#### 2.4.7. Determination of Fatty Acid Profile

Fatty acid methyl esters (FAME) were obtained from intramuscular fat (previously extracted) by methylation using. KOH (85% in MetOH). Separation of FAMEs was carried out using a gas chromatograph (model 4890 Series II; Hewlett-Packard, Palo Alto, CA, USA) fitted a Carbowax™ fused silica capillary column (30 m × 0.25 mm id; 0.25 μm film thickness; (Ohio Valley, Marietta, OH, USA). For the identification of individual FAME was used by a standard mixture of 37 Component FAME Mix (Sigma–Aldrich, Supelco 37 Component FAME Mix- CRM47885, St. Louis, MO, USA).

#### 2.4.8. Texture Analysis

Prior to fresh loin texture assays samples were vacuum-packing in nylon/polyethylene bags, and cooking by immersion at 80 °C for 45 min in a water bath preheated at until an internal temperature of 75 °C was reached. Texture analysis was performed in a texturometer TA XT-2i Texture Analyser (Stable Micro Systems Ltd., Surrey, UK).

Two texture analysis were carried out: Texture Profile Analysis (TPA) and Warner-Bratzler test (WBSF).

TPA was determined at two deformation percentages; (i) TPA I (20%) in order to evaluate the contribution of myofibrillar structures and (ii) TPA II (80%) to analyze the intervention of the connective tissue on to textural properties [20]. For the determination of TPA, each sample was cut into uniform cubes of 1 cm^3^ and which were compressed in a parallel direction to the muscle fibers to 20% (TPA I) and 80% (TPA II) of the original height with a flat plunger of 20 mm diameter (P/20) using a 25-kN load cell applied at a crosshead speed of 2 mm/s through a 2-cycle sequence. The following texture parameters were measured from force–deformation curves [21]; hardness (N/cm^2^), springiness (cm), cohesiveness (dimensionless), gumminess (N cm s^2^), chewiness (N cm s^2^), and resilience (dimensionless), as mean values. TPA analysis in dry-cures loin was performing with a 50% deformation [22].

Both cooked and dry-cured loins were cutted 1 mm^2^ cross-sections with the muscle fibers parallel to the longitudinal axis and which were cut with a Warner–Bratzler device (HDP/BS) in perpendicular direction to the muscle fibers. The maximum shear force to cut the sample was measured. Instrumental determinations were repeated 8 times per sample and results were data averaged. Results were expressed as WBSF (N/cm^2^).

#### 2.4.9. Statistical Analysis

The effects of freezing and pre-freezing cure time (0, 3, and 6 months) on fresh and dry-cured loin respectively, were evaluated on proximate composition, water losses, instrumental colour, antioxidant, oxidative status, fatty acid profile, and textural properties by one-way analysis of variance (ANOVA) using SPSS version 20.0 (SPSS Inc,. Chicago, IL, USA). Each loin (raw, frozen/thawed, or dry-cured) was considered as one unit. Data are presented as mean values ± standard error for each group (LLU, LLF3, and LLF6 for raw and frozen/thawed loins and DCU, DCF3, and DCF6 for dry-cured and pre-frozen dry-cured loins, respectively). Statistical significance was assessed by Tukey’s HSD test, and the level of signification was set at *p* = 0.05. The significant differences post-hoc test was used to compare groups.

## 3. Results and Discussion

### 3.1. Effect of Pre-Freezing Cure Time on Proximate Composition, Water Losses and Instrumental Colour

Freezing did not significantly affect proximate composition of fresh loins (Table 1) (*p* > 0.05). With regard to water losses (Table 1), freezing storage time did not influence this parameter—on fresh loin— (*p* > 0.05), but differences in the capacity of the muscle to retain water were observed (*p* ≤ 0.001). In this respect, there is a general agreement regarding the formation of relatively long and irregular ice crystals in conventional freezing (−20 °C). This could have led to damaging the structure of the LLF3 and LLF6, resulting in a loss of water after freezing and thawing process [23]. This phenomenon might have been the reason of the lower volume of water available to be released when an external force was exerted in LLF3 and LLF6 with respect to LLU (Table 1) (*p* ≤ 0.001). Our results are in line with Kim et al. [24], who observed less released water after freezing/thawing of m. *Longissimus thoracis et lumborum* from pigs when measuring cooking losses.

The higher WL observed in pre-frozen dry-cured loins (DCF3 and DCF6) (*p* ≤ 0.001) (Table 1) could be therefore explained by the easier release of water from the meat piece during curing because of a breakdown of the muscle fibers and subsequent loss of structural integrity caused by ice crystal formation. Nonetheless, similar studies carried out on Iberian dry-cured hams observed no differences in this parameter accounting on pre-freezing cure [3,25]. Discrepancies between studies may be related with proximate composition—especially the amount of IMF, higher in hams than loins, which could have overcome the possible differences exerted by pre-freezing cure practice. Moreover, Iberian dry-cured hams are subjected to a longer dry-curing process, and therefore the dynamics of water may differ with respect to dry-cured loins. Therefore, the influence of pre-freezing cure could affect differently to dry-cured ham and loin. The higher values of WL in pre-frozen dry-cured loins (DCF3 and DCF6) possibly led to their lower moisture content (*p* ≤ 0.001) (Table 1). Therefore, pre-freezing cure could allow the production of *Montanera* Iberian loin to be adjusted to consumer demand, given that the minimum length required for curing is regulated by the IQS [2], and cannot be less than 70 days. However, this practice might cause a lower performance of the pieces, which would lead to greater economic losses as a result of the cost of the freezing process and the lower final weight of the pre-frozen dry-cured loins.

The lower values found in NaCl content in DCF3 and DCF6 with respect to DCU (*p* ≤ 0.001) (Table 1), could be closely connected with the fact that pre-frozen dry-cured loins showed a lower moisture content (*p* ≤ 0.05). This fact could have led to a lower diffusion of salt within the loin. This hypothesis is in line with the results found by Pérez-Álvarez, Sayas-Barberá, Fernández-López, Gago-Gago, Pagán-Moreno, and Aranda-Catalá [26] in dry-cured hams. Conversely Abellán et al. [11] and Pérez-Palacios et al. [3] reported an opposite pattern for this parameter, attaining higher salt content in pre-frozen dry-cured loins and hams, respectively, when they were compared to those from unfrozen counterparts. The penetration of salt also is dependent on other factors such as muscle structure. Differences in this latter because of the different conditions of the process of freezing/thawing [11] may be responsible of the discrepancies in results of salt content among studies. It is therefore important to identify the characteristics of the process of freezing/thawing of raw material previous to the technological process of curing, as this could have an impact during salting/seasoning. Unfortunately, references on the effect that the frozen storage of the raw muscle has on salt content in dry-cured loin is scarce [11], and inexistent when it comes to Iberian dry-cured loin.

Regarding instrumental colour coordinates, freezing and thawing showed a significant effect on lightness (L*), redness (a*), chroma (C*) and hue angle values (H°) (*p* ≤ 0.001) of fresh loins, regardless of storage time (*p* > 0.05). Contrarily, no effect was observed for yellowness values (b*) (*p* > 0.05).

The lower L* values in LLF3 and LLF6 are in line with results by Kim et al. [24], and could be ascribed to their higher water losses as compared to LLU, as a result of freezing/thawing (Table 1). Freezing and thawing also promoted a decrease in a* and C* values, as well as an increase in H° values leading to a decrease in the intensity of the red colour in LLF3 and LLF6. Our results are in concordance with those by Martín-Mateos [27], who found a decrease in the red colour of frozen and thawed m. *Serratus ventralis* from Iberian pigs after 18 months of freezing storage. Similarly, Bañón et al. [8] observed a slight decrease in a* in frozen/thawed commercial hams. On the contrary, Pérez-Palacios et al. [3] found higher values of a* and C* in frozen/thawed Iberian hams, when they were compared to unfrozen ones, which was attributed to a higher level of desiccation.

No differences were observed for b* on fresh loin after freezing. There is a wide range of results within the existing scientific literature. Whereas Bañón et al. [8] described a reduction of b* values as a result of freezing in commercial hams, Pérez-Palacios et al. [3] did not observed any difference in Iberian hams pieces. Similarly, Kim et al. [24], did not observe changes in b* coordinates in m. *Longissimus thoracis et lumborum* from commercial breed as a result of freezing/thawing process.

The discrepancies noted for some of the coordinates studied among results from the current study and those dealing with pre-freezing cure in hams could be attributed not only to differences in chemical composition between products, but also to the influence of the component distribution on instrumental colour, as concluded Carrapiso and García [28].

Despite the differences in instrumental colour observed in the raw material due to freezing, these differences are not evident for L*, a* and C* values after curing process (*p* > 0.05) (Table 1). This is consistent with previous studies in Iberian products, where the instrumental colour differences found in cured meat were not exactly alike in the fresh meat [28].

On the opposite, differences in b* were found in dry-cured loin because of the pre-freezing cure, with DCU yielding the highest value (*p* ≤ 0.001), which led to a decrease in H° (*p* ≤ 0.001) (Table 1). This could be attributed to the lower moisture and salt content of pre-frozen dry-cured loins [26].

### 3.2. Effect of Pre-Freezing Cure Time on Antioxidant Content, Oxidative Status, and Fatty Acids Profile

As far as antioxidants content in fresh loin is concerned, no differences were observed due to freezing/thawing neither to freezing storage time (*p* > 0.05) (Table 2). The values of α- and γ-tocopherol are influenced by the feeding of the animals. In particular, in the meat from Iberian pigs fattened in *Montanera*, the α- and γ- tocopherol content is attributed to the intake of grass and acorns. [29]. The lack of differences in tocopherol content between LLU and LLF3 and LLF6 could be explained by the stability of these compounds. In fact, previous studies have reported a low α-tocopherol degradation in Iberian meat after 18 months of freezing storage [27]. Contrariwise, this latter author reported a degradation of γ-tocopherol in the muscle *Serratus ventralis* after 12 months of freezing storage. In any case, the time of freezing storage studied in our research was shorter, which could explain the lack of degradation of γ-tocopherol in both LLF3 and LLF6.

On the contrary, differences in γ-tocopherol content were found among loin sets after curing (*p* ≤ 0.001) (Table 2). It can be observed that both α- and γ-tocopherol attained higher values in pre-frozen dry-cured loins (DCF3 and DCF6) with respect to DCU, although only significant differences were found for the latest. This finding could be explained by their slightly higher IMF, since all fresh loin sets started the curing process with homogenous values. Our results suggest that a great stability of tocopherols could be predicted not only during curing process, as previous studies have reported [30,31], but also after pre-freezing cure.

Lipid oxidation (Table 2) increased (*p* ≤ 0.001) after freezing/thawing. This trend is in agreement with Martín Mateos [27] and Lorido et al. [10] who found higher oxidation degree after twelve and five months of freezing storage of *Serratus ventralis* and *Longissimus dorsi* respectively, in comparison with unfrozen ones. Likewise, freezing and thawing process, as well as freezing storage time seem to have promoted a higher protein oxidation (*p* ≤ 0.001) (Table 2), which is associated with a decrease in thiol groups [4,10]. This increase in protein oxidation is consistent with studies carried out by other authors done on Iberian meat [10,27] and could be related to lipid oxidation, since previous studies have reported that products from lipid oxidation may serve as a substrate for protein oxidation [6].

The trend observed for oxidative status remained similar after curing, pre-frozen dry-cured loin (DCF3 and DCF6) showing the highest oxidation values, probably explained by their higher initial values (Table 2). With respect to lipid oxidation, results obtained in this study are in disagreement with those obtained by Pérez-Palacios et al. [5] and Lorido et al. [10], who did not observe any impact of pre-freezing cure on lipid oxidation of Iberian dry-cured hams and loins, respectively. Differences in results among scientific literature could be related to the fatty acid composition, which affects oxidative stability of dry-cured products. Thus, fat from Iberian hams or loins from animals fed with a commercial diet yield high levels of SFA, being a fat with high consistency, and therefore being more stable than fat from *Montanera* pigs, in which the high level of PUFA make [32] it more prone to oxidative degradation [33]. These results would suggest the influence of the productive system influences on the effect exerted by the pre-freezing cure. Results obtained in this study for protein oxidation are in agreement with Lorido et al. [10] who reported an increase in protein oxidation of dry-cured loins from frozen/thawed pieces with respect to unfrozen ones.

Regarding fatty acid profile (Table 2), PUFA content was lower in frozen/thawed loins (LLF3 and LLF6) (*p* ≤ 0.001), mainly explained by the decrease in C18:2 n-6 and C18:3 n-3 (*p* ≤ 0.05), which led to a higher n-6/n-3 relationship (*p* ≤ 0.001). It could be hypothesized that the lower content in PUFA in LLF3 and LLF6 after freezing/thawing could be ascribed to its oxidation, which was more intense in these samples (Table 2) (*p* ≤ 0.001) and considering that it is generally accepted that oxidation phenomena is initiated by the autooxidation of PUFA [4]. In fact, higher lipid oxidation values were observed in these samples –LLF3 and LLF6- (Table 2) (*p* ≤ 0.001). Our findings are in agreement with Hernández, Navarro, and Toldrá [34] and Perez-Palacios et al. [5] who observed a decrease in PUFA in loin from commercial pig breed and on the polar lipid fraction of Iberian hams after freezing storage.

The differences in the fatty acid profile observed for fresh loin were maintained after curing (*p* ≤ 0.05) (Table 2). Our results disagree with those found by Pérez-Palacios et al. [5], who observed no difference in the fatty acid profile of Iberian dry-cured hams accounting on pre-freezing cure. However, to our knowledge there are no studies in Iberian dry-cured loins to contrast our results.

### 3.3. Effect of Pre-Freezing Cure Time on Textural Properties

As can be observed in Table 3, freezing affected most of the textural parameters evaluated on fresh loin. More specifically, results from TPA I showed that loins under freezing storage (LLF3 and LLF6) yielded higher hardness, gumminess, and chewiness than unfrozen ones (*p* ≤ 0.001). On the contrary, freezing storage time did not exert any effect on these parameters. Contrarily, a previous study by Pérez-Palacios et al. [3] showed a decrease in hardness and chewiness—in a 50% compression test—in Iberian hams prior to curing after three months of freezing storage.

However, loin is a different piece, with a lower IMF and therefore it could be more sensitive to water loss, which is related to tenderness [35].

Regarding TPA II, LLF3 and LLF6 showed higher values for hardness, springiness, cohesiveness, gumminess, and chewiness (*p* ≤ 0.001) than unfrozen ones, but no differences were found in resilience (*p* > 0.05) with respect to LLU. As it was observed for TPA I, no differences accounting on freezing storage time were observed. The higher hardness after freezing/thawing in tests with high percentages of deformation could be justified with the greater degree of protein oxidation, specially of the connective tissue [36]. Our results are in agreement with Martín-Mateos [27], who observed an increase in gumminess, chewiness and hardness in high compression test in *Serratus ventralis* after 365 days frozen.

With respect to WBSF test, LLF3 and LLF6 yielded lower values than those showed by LLU (*p* ≤ 0.001). These results could be due to the rupture of muscle fibers, as well as by the loss of cell membrane resistance after freezing and thawing process. Martín-Mateos [27] also observed the same trend in shear force though in *Serratus ventralis* after one year of freezing storage.

The pre-freezing cure, DCF3 and DCF6, kept maintaining the higher values in hardness, springiness, and chewiness (*p* ≤ 0.05), when a deformation test (TPA 50%) was applied to them (Table 4), whilst resilience values of DCF3 and DCF6 with respect to DCU decreased (*p* ≤ 0.001). No differences were observed for cohesiveness and gumminess in dry-cured loin on account of pre-freezing cure (*p* > 0.05). More specifically, the higher values in hardness for DCF3 and DCF6 could be explained by several factors: (i) by the higher hardness of frozen/thawed raw material (*p* ≤ 0.001) (Table 3), (ii) by the increase in protein oxidation, which was more intense in pre-frozen dry-cured loins (*p* ≤ 0.001) (Table 2) [23] and (iii) by the higher WL (*p* ≤ 0.001) (Table 1) [37] to which these pieces were subjected during curing. Our results agree with Lorido et al. [10], who observed higher hardness in Iberian dry-cured loins from frozen/thawed pieces than unfrozen ones, measured by a sensory panel. Regarding WBSF, pre-frozen dry-cured loins (DCF3 and DCF6) yielded lower values than DCU (*p* ≤ 0.001) (Table 4), imitating the pattern previously observed in fresh loin (*p* ≤ 0.001) (Table 3).

With respect to scientific literature, Pérez-Palacios et al. [3] reported a lack of differences in instrumental texture accounting on pre-freezing cure on Iberian dry-cured hams, suggesting that the technological process might balance texture differences found in fresh meat. However, no studies dealing with this topic on dry-cured loin were found.

## 4. Conclusions

Our results suggest that pre-freezing cure (three and six months) on dry-cured loins from Iberian x Duroc pigs reared under *Montanera* system had no effect on some quality traits such as lightness, redness, α-tocopherol and MUFA. However, differences were observed in parameters related to PUFA content, oxidative status, and textural properties (mainly hardness).

From these results it can be also inferred that quality and technological aptitude of raw material (frozen/thawed vs. unfrozen) had an important effect on weight loss during curing, which could affect the productive and economic performance of the meat industry. Hence, it would be recommendable the pre-freezing cure practice to be considered as a feasible practice by the current Iberian Quality Standard, in order to adjust curing conditions to the type of meat (fresh or pre-freezing).

The present research was intended as a preliminary study to provide insight into Iberian meat industry. Further researches should be carried out to assess the influence of this practice on shelf life and to explore whether physical–chemical differences are perceived by consumers and therefore the impact in their purchasing decisions.

## Figures and Tables

**Table 1:** Effect of freezing and pre-freezing cure on proximate composition, water losses, and instrumental colour.

	Fresh Loin				Dry-Cured Loin			
	LLU	LLF3	LLF6	Sig	DCU	DCF3	DCF6	Sig
Proximate composition (g/100 g)								
Moisture	70.1 ± 1.28	70.7 ± 0.70	70.1 ± 0.74	ns	44.2 ^a^ ± 2.09	41.9 ^b^ ± 3.21	42.4 ^b^ ± 2.28	*
IMF ^1^	12.1 ± 1.08	13.5 ± 1.55	12.7 ± 2.75	ns	16.4 ± 1.08	17.6 ± 0.97	17.3 ± 2.44	ns
NaCl	-	-	-		5.6 ^a^ ± 0.66	4.1 ^b^ ± 0.43	4.1 ^b^ ± 0.50	***
Water losses (g water/100 g muscle)								
TL	-	2.5 ± 0.54	2.7 ± 0.62	ns	-	-	-	
WL	-	-	-		37.6 ^c^ ± 1.78	39.2 ^b^ ± 1.47	41.3 ^a^ ± 1.97	***
WHC	29.0 ^a^ ± 0.50	26.4 ^c^ ± 1.39	28.0 ^b^ ± 0.46	***	-	-	-	-
Instrumental Color coordinates								
L *	47.9 ^a^ ± 2.48	44.9 ^b^ ± 2.51	45.4 ^b^ ± 2.67	***	35.3 ± 2.17	36.7 ± 3.87	36.3 ± 3.29	ns
a *	14.0 ^a^ ± 1.51	12.5 ^b^ ± 1.20	12.0 ^b^ ± 1.24	***	12.6 ^a^ ± 1.04	11.0 ^b^ ± 0.50	12.6 ^a^ ± 0.85	ns
b *	7.5 ± 1.28	7.4 ± 0.70	7.5 ± 0.54	ns	6.3 ^a^ ± 0.54	4.5 ^b^ ± 0.19	3.5^c^ ± 0.39	***
C *	16.2 ^a^ ± 1.70	14.3 ^b^ ± 1.47	13.8 ^b^ ± 1.66	***	14.2 ± 1.12	12.0 ± 0.93	12.8 ± 0.85	ns
H°	20.0 ^b^ ± 1.32	30.0 ^a^ ± 1.55	30.4 ^a^ ± 1.20	***	26.3 ^a^ ± 1.47	22.0 ^a^ ± 2.05	15.4 ^b^ ± 1.08	***

Values are expressed as means ± standard error of 15 samples/treatment. ^1^ Expressed in grams per 100 g moisture-free tissue. Effect: freezing time on LLU, LLF3, and LLF6 = unfrozen, and frozen during three- or six-months *Longissimus lumborum* muscle, respectively and pre-freezing cure time on DCU, DCF3 and DCFF6 = dry-cured loins from *Longissimus thoracis* muscle unfrozen and frozen during three and six months, respectively. IMF, intramuscular fat; NaCl, salt content; TL, thawing loss; WL, weight loss during curing process; WHC, water holding capacity. Values with the same letters (a, b, c) indicate homogeneous subsets for *p* = 0.05 according to Tukey’s HSD test. ns: (*p* > 0.05); * (*p* ≤ 0.05); *** (*p* ≤ 0.001).

**Table 2:** Effect of freezing and pre-freezing cure on antioxidant content, oxidative status, and fatty acids profile.

	Fresh Loin				Dry-Cured Loin			
	LLU	LLF3	LLF6	Sig	DCU	DCF3	DCF6	Sig
Antioxidant Composition and Oxidative status								
α-Tocopherol ^1^	16.4 ± 1.43	16.6 ± 2.28	16.3 ± 2.21	ns	10.7 ± 0.89	11.1 ± 2.67	11.7 ± 0.97	ns
γ-Tocopherol ^1^	2.6 ± 0.43	2.9 ± 0.50	2.9 ± 0.46	ns	1.6 ^b^ ± 0.31	1.9 ^a^ ± 0.38	2.0 ^a^ ± 0.31	**
mg MDA/kg	0.1 ^b^ ± 0.04	0.2 ^a^ ± 0.08	0.2 ^a^ ± 0.04	***	1.0 ^b^ ± 0.19	1.3 ^a^ ± 0.31	1.2 ^a^ ± 0.36	*
nmol thiol/mg prot	166.7 ^a^ ± 21.98	127.4 ^b^ ± 14.20	88.1 ^c^ ± 16.69	***	210.0 ^a^ ± 16.87	147.8 ^b^ ± 33.59	138.8 ^b^ ± 16.18	***
Fatty acid composition (g/100 g FAMEs)								
C16:0	23.0 ± 1.20	23.6 ± 1.78	23.4 ± 2.32	ns	23.8 ± 2.05	23.5 ± 1.27	23.9 ± 1.90	ns
C16:1	3.8 ± 0.35	3.9 ± 0.43	3.8 ± 0.69	ns	3.9 ± 0.69	3.7 ± 0.38	3.8 ± 0.34	ns
C18:0	11.0 ± 0.81	10.3 ± 1.12	10.8 ± 1.54	ns	11.6 ± 1.78	12.2 ± 0.97	11.2 ± 1.63	ns
C18:1 n-9	52.1 ± 1.39	52.1 ± 0.77	52.3 ± 1.35	ns	51.6 ± 2.83	50.3 ± 1.89	51.0 ± 1.32	ns
C18:2 n-6	6.2 ^a^ ± 0.31	6.0 ^a b^ ± 0.54	5.6 ^b^ ± 0.81	*	5.6 ^a b^ ± 0.62	5.7 ^a^ ± 0.43	5.2 ^b^ ± 0.54	*
C18:3 n-3	0.5 ^a^ ± 0.01	0.3 ^b^ ± 0.04	0.4 ^b^ ± 0.07	***	0.3 ^a^ ± 0.07	0.2 ^b^ ± 0.04	0.2 ^b^ ± 0.04	***
SFA	35.5 ± 1.90	36.4 ± 2.36	36.7 ± 3.72	ns	36.8 ± 3.98	37.9 ± 2.39	38.0 ± 2.59	ns
MUFA	56.9 ± 1.66	56.9 ± 0.93	56.9 ± 1.74	ns	56.2 ± 1.89	56.0 ± 2.01	56.1 ± 1.35	ns
PUFA	7.7 ^a^ ± 0.43	6.7 ^b^ ± 0.58	6.4 ^b^ ± 0.85	***	7.1 ^a^ ± 0.74	6.1 ^b^ ± 0.74	5.9 ^b^ ± 0.54	***
n-6/n-3	11.9 ^c^ ± 0.77	18.3 ^a^ ± 2.28	16.4 ^b^ ± 2.78	***	17.6 ^b^ ± 2.47	22.5 ^a^ ± 3.63	21.9 ^a^ ± 2.40	***

Values are expressed as means ± standard error of 15 samples/treatment. ^1^ Expressed as µg of α- or γ-tocopherol/g moisture-free tissue. Effect: freezing time on LLU, LLF3 and LLF6 = unfrozen, and frozen during three- or six-months *Longissimus lumborum* muscle, respectively and pre-freezing cure time on DCU, DCF3 and DCFF6 = dry-cured loins from *Longissimus thoracis* muscle unfrozen and frozen during three and six months, respectively. FAMEs, fatty acid methyl esters; C16:0, palmitic acid; C16:1, palmitoleic acid; C18:0, stearic acid; C18:1 n-9, oleic acid; C18:2 n-6, linoleic acid, C18:3 n-3, linolenic acid, SFA: sum of all saturated fatty acids detected (C14:0, C16:0, C17:0; C18:0, C20:0); MUFA: sum of all monounsaturated fatty acids detected (C16:1, C17:1, C18:1, C20:1); PUFA: sum of all polyunsaturated fatty acids detected (C18:2 n-6, C18:3 n-3; C20:2 n-9; C20:3 n-6; C20:4 n-6; C20:3 n-3); n-6/n-3: PUFA n-6/PUFA n-3 ratio. Values with the same letters (a, b, c) indicate homogeneous subsets for *p* = 0.05 according to Tukey’s HSD test. ns: (*p* > 0.05); * (*p* ≤ 0.05); ** (*p* ≤ 0.01); *** (*p* ≤ 0.001).

**Table 3:** Effect of freezing on textural properties of fresh loin.

	Fresh Loin			
	LLU	LLF3	LLF6	Sig
Compression Test I (TPA-20% compression)				
Hardness (N/cm^2^)	1.9 ^b^ ± 0.50	5.2 ^a^ ± 1.47	5.5 ^a^ ± 1.47	***
Springiness (cm)	0.8 ± 0.04	0.8 ± 0.04	0.9 ± 0.04	ns
Cohesiveness	0.7 ± 0.04	0.7 ± 0.04	0.7 ± 0.04	ns
Gumminess (N cm s^2^)	1.2 ^b^ ± 0.19	3.7 ^a^ ± 1.16	4.1 ^a^ ± 1.16	***
Chewiness (N cm s^2^)	1.0 ^b^ ± 0.27	3.0 ^a^ ± 1.08	3.5 ^a^ ± 1.08	***
Resilience	0.5 ^b^ ± 0.04	0.5 ^a^ ± 0.08	0.5 ^a^ ± 0.04	ns
Compression Test II (TPA-80% compression)				
Hardness (N/cm^2^)	100.0 ^b^ ± 10.02	134.3 ^a^ ± 12.93	132.1 ^a^ ± 12.03	***
Springiness (cm)	0.5 ^b^ ± 0.04	0.5 ^a^ ± 0.04	0.5 ^a^ ± 0.04	***
Cohesiveness	0.4 ^b^ ± 0.04	0.5 ^a^ ± 0.04	0.5 ^a^ ± 0.04	***
Gumminess (N cm s^2^)	41.4 ^b^ ± 8.05	61.6 ^a^ ± 11.14	62.8 ^a^ ± 12.54	***
Chewiness (N cm s^2^)	17.8 ^b^ ± 4.02	32.7 ^a^ ± 6.85	33.3 ^a^ ± 7.93	***
Resilience	0.2 ± 0.04	0.3 ± 0.04	0.3 ± 0.04	ns
Warner—Braztler shear force test (WBSF)				
WBSF (N/cm^2^)	72.0 ^a^ ± 13.43	46.2 ^b^ ± 9.79	49.3 ^b^ ± 11.22	***

Values are expressed as means ± standard error of 15 samples/treatment. Effect: freezing time: LLU, LLF3, and LLF6 = unfrozen, and frozen during three- or six-months *Longissimus lumborum* muscle, respectively. TPA, textural profile analysis. Values with the same letters (a, b) indicate homogeneous subsets for *p* = 0.05 according to Tukey’s HSD test. ns: (*p* > 0.05); *** (*p* ≤ 0.001).

**Table 4:** Effect of pre-freezing cure on textural properties of dry-cured loins.

	Dry-Cured Loin			
	DCU	DCF3	DCF6	Sig
Compression Test (TPA-50% compression)				
Hardness (N/cm^2^)	30.9 ^b^ ± 2.97	33.2 ^a b^ ± 1.70	36.0 ^a^ ± 2.36	*
Springiness (cm)	0.6 ^b^ ± 0.04	0.7 ^a^ ± 0.08	0.7 ^a^ ± 0.04	***
Cohesiveness	0.6 ± 0.04	0.6 ± 0.04	0.6 ± 0.04	ns
Gumminess (N cm s^2^)	18.5 ± 4.91	20.1 ± 3.44	21.8 ± 3.64	ns
Chewiness (N cm s^2^)	10.7 ^b^ ± 3.02	13.9 ^a^ ± 1.97	14.2 ^a^ ± 2.17	***
Resilience	2.1 ^a^ ± 0.31	0.2 ^b^ ± 0.04	0.2 ^b^ ± 0.04	***
Warner–Braztler shear force test				
WBSF (N/cm^2^)	44.1 ^a^ ± 5.68	33.5 ^b^ ± 5.46	32.6 ^b^ ± 3.48	***

Values are expressed as means ± standard error of 15 samples/treatment. Effect: Pre-freezing cure time: DCU, DCF3 and DCFF6 = dry-cured loins from *Longissimus thoracis* muscle unfrozen and frozen during three- or six-months, respectively. TPA, textural profile analysis. Values with the same letters (a, b) indicate homogeneous subsets for *p* = 0.05 according to Tukey’s HSD test. ns (*p* > 0.05); * (*p* ≤ 0.05); *** (*p* ≤ 0.001).

## Data Availability

Data sharing not applicable.
